# Polarization of Macrophages in Insects: Opening Gates for Immuno-Metabolic Research

**DOI:** 10.3389/fcell.2021.629238

**Published:** 2021-02-15

**Authors:** Adam Bajgar, Gabriela Krejčová, Tomáš Doležal

**Affiliations:** Department of Molecular Biology and Genetics, University of South Bohemia, Ceske Budejovice, Czechia

**Keywords:** *Drosophila*, macrophages, insulin resistance, cachexia, cytokines, immuno-metabolism, aerobic glycolysis

## Abstract

Insulin resistance and cachexia represent severe metabolic syndromes accompanying a variety of human pathological states, from life-threatening cancer and sepsis to chronic inflammatory states, such as obesity and autoimmune disorders. Although the origin of these metabolic syndromes has not been fully comprehended yet, a growing body of evidence indicates their possible interconnection with the acute and chronic activation of an innate immune response. Current progress in insect immuno-metabolic research reveals that the induction of insulin resistance might represent an adaptive mechanism during the acute phase of bacterial infection. In *Drosophila*, insulin resistance is induced by signaling factors released by bactericidal macrophages as a reflection of their metabolic polarization toward aerobic glycolysis. Such metabolic adaptation enables them to combat the invading pathogens efficiently but also makes them highly nutritionally demanding. Therefore, systemic metabolism has to be adjusted upon macrophage activation to provide them with nutrients and thus support the immune function. That anticipates the involvement of macrophage-derived systemic factors mediating the inter-organ signaling between macrophages and central energy-storing organs. Although it is crucial to coordinate the macrophage cellular metabolism with systemic metabolic changes during the acute phase of bacterial infection, the action of macrophage-derived factors may become maladaptive if chronic or in case of infection by an intracellular pathogen. We hypothesize that insulin resistance evoked by macrophage-derived signaling factors represents an adaptive mechanism for the mobilization of sources and their preferential delivery toward the activated immune system. We consider here the validity of the presented model for mammals and human medicine. The adoption of aerobic glycolysis by bactericidal macrophages as well as the induction of insulin resistance by macrophage-derived factors are conserved between insects and mammals. Chronic insulin resistance is at the base of many human metabolically conditioned diseases such as non-alcoholic steatohepatitis, atherosclerosis, diabetes, and cachexia. Therefore, revealing the original biological relevance of cytokine-induced insulin resistance may help to develop a suitable strategy for treating these frequent diseases.

## Introduction

Both cachexia and insulin resistance are in the spotlight of immuno-metabolic research and represent the most important comorbidities that often accompany acute and chronic inflammatory states and complicate their treatment (Fonseca et al., 2020). Cachexia, literally meaning “bad condition,” is a metabolic syndrome of excessive weight loss and muscle wasting caused by alterations in appetite and the overall metabolic setup (Yang et al., 2020). The progressive development of insulin resistance to pre-cachexia and cachexia, which is defined as a loss of more than 5% of the cell body mass over 12 months or less, is known to be a hallmark for a wide range of seemingly unrelated diseases, such as obesity, cancer, chronic obstructive pulmonary disease, acute kidney disease, and sepsis (Mak and Cheung, 2006; Koehler et al., 2007; Srikanthan et al., 2010; Honors and Kinzig, 2012). Nevertheless, the mechanism of induction of these frequently occurring metabolic syndromes remains to be elucidated.

The origin of insulin resistance and cachexia relies on the activity of immune cell-derived signaling factors and is thus a result of excessive activation of the immune system (Olefsky and Glass, 2010). However, the biological relevance of such signaling has not been fully comprehended yet. It is mainly due to the prevailing perception of the cytokine-induced insulin resistance as a mere side effect of pathological syndromes and insufficient effort to reveal its adaptive meaning. The complexity of the mammalian immune system, as well as pleiotropic effects of most immune cell-derived factors, further complicate the resolution of this intricate relationship (Stenholm et al., 2008; Del Fabbro et al., 2011).

Recent progress in insect immuno-metabolic research revealed that cytokine-induced insulin resistance is not a mechanism occurring exclusively in vertebrates. Indeed, we may observe several physiological conditions in which immune cells release cytokines to affect the systemic metabolism via induction of insulin resistance in *Drosophila*, such as metabolic misbalance and development, as well as immune response (Rajan and Perrimon, 2011; Woodcock et al., 2015; Lee et al., 2018; Dolezal et al., 2019). These states document the preservation of this mechanism among such evolutionarily distant groups as insects and mammals. To be maintained in the evolution, we might presume that cytokine-induced insulin resistance represents an ancient adaptive process of systemic metabolic rearrangement.

**FIGURE 1:**
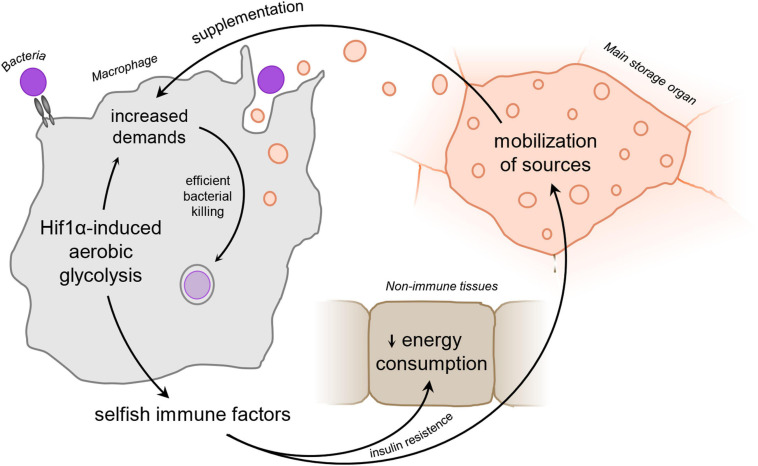
Schematic representation of the “selfish immune system theory.” Infection-activated macrophages adopt Hif1α-induced aerobic glycolysis and subsequently release signaling factors to ensure sufficient amount of nutrients to supplement the immune function. Hif1α, hypoxia-inducible factor 1 α.

Here, we would like to present several recent observations depicting that *Drosophila* activated immune cells affect systemic metabolism via the induction of insulin resistance to ensure sufficient supplementation with nutrients for their function (Figure 1 and Box 1). Although this mechanism is necessary for the acute phase of the immune response (Yang et al., 2015; Bajgar and Dolezal, 2018; Dolezal et al., 2019), it may lead to nutrient wastage if chronic, and prolonged reallocation of sources may become the basis for the development of many serious pathological conditions.

**BOX 1. Hypothesis.**

We hypothesize that activated phagocytes produce signaling factors to reflect their current nutritional demands upon adoption of aerobic glycolysis. These factors induce mobilization of nutrients and silence their consumption by non-immune tissues via insulin resistance, leaving thus enough of sources for the activated immune system. Release of these signaling factors is thus beneficial for the acute immune response; however, it may lead to energy wasting and development of severe pathologies if produced chronically (Figure 1).

Innate immune cells performing the phagocytic function represent the front line of protection against invading pathogens (Franken et al., 2016). Individuals, therefore, tend to maximize the number of these protectors participating in phagocytosis and clearance of pathogen (Kacsoh and Schlenke, 2012; Mihajlovic et al., 2019). Nevertheless, the maintenance of an excessive number of metabolically demanding phagocytes would be highly energy-intensive with subsequent adverse impact on concurrent energy-consuming processes, such as growth and reproduction (Wolowczuk et al., 2008). Therefore, animals have developed a strategy to overcome these evolutional constraints by maintaining a sufficient number of immune cells in a quiescent state as well as by proliferation of their progenitors upon immune challenge. Quiescent phagocytes exhibiting only a basal metabolic rate are thus waiting for the activation stimuli ready to be metabolically awakened and to participate in the acute immune response (Mosser and Edwards, 2008). In plentiful times, individuals can fully exploit the surplus energy to maintain homeostasis, growth, and reproduction as processes based mainly on anabolic metabolism (Wang et al., 2019). That is in sharp contrast to the situation of life-threatening infection. In response to the recognition of pathogen-associated molecular patterns, activated immune cells such as monocytes, macrophages, dendritic cells, and neutrophils, must react rapidly to limit the pathogen burden and adopt a bactericidal polarization phenotype (Galván-peña and O’Neill, 2014; Loftus and Finlay, 2016). However, the immediate activation of a large number of these cells toward the bactericidal phenotype (also known as pro-inflammatory) represents an immense energy load for the organism (Demas, 2004; Edholm et al., 2017). The nutritional investments connected with the acute phase response are further increased by the proliferation of immune cell progenitors and their differentiation toward effector cells upon activation of the immune response.

Professional phagocytes must rewire their cellular metabolism greatly to become efficient in bacterial killing (Pavlou et al., 2017). It is well established particularly for mammalian bactericidal macrophages that they undergo metabolic polarization toward aerobic glycolysis as a predominant source of energy and a precursors essential for bactericidal function (Benoit et al., 2008). Similarly to macrophages, the increased glycolytic rate and other metabolic adjustments were later confirmed also for neutrophils, dendritic cells, effector lymphocytes, and natural killer cells (Loftus and Finlay, 2016). Interestingly, adoption of aerobic glycolysis by immune cells may originate not only in response to bacterial invaders but can also be induced by excessive lipid uptake (Box 2).

**BOX 2. Excessive lipids induce adoption of macrophage pro-inflammatory phenotype.**

It is of particular interest that the adoption of pro-inflammatory Ml polarization can be induced even without the presence of a pathogen. That is in concordance with the previously mentioned fact that HIFlα stabilization, central for induction of bactericidal macrophage polarization, may be achieved either by TLR4 activation or by metabolic feedback from mitochondrial metabolism (Iommarini et al., 2017). It underpins many metabolically induced inflammatory diseases with a significant impact on human well-being, such as obesity, non-alcoholic fatty liver disease, atherosclerosis, and diabetes (Kraakman et al., 2014; Castoldi et al., 2016; Kazankov et al., 2019).

Exposure of macrophages to excessive amounts of lipids can lead to the adoption of pro-inflammatory polarization of macrophages. The effect of lipids on macrophages is dual. The increased concentration of lipids in the extracellular space is recognized by TLR4 and, analogically to infection, leads to the stabilization of HIFlα via the NFKB signaling pathway (Hubler and Kennedy, 2016; Korbecki and Bajdak-Rusinek, 2019). In addition, lipids are efficiently internalized by macrophages via receptor-mediated endocytosis (Park, 2014). Because there is no feedback on lipid uptake by macrophages, it leads to a massive accumulation of oxidized lipids and cholesterol in the cytosol of these cells, followed by disruption of mitochondrial function (Gibson et al., 2018). Lipid peroxidation catalyzed by free iron ions, together with ROS accumulation, leads to disruption of mitochondrial function by activating the transcription factor *nuclear factor erythroid 2-related factor 2* (NRF2) (Dodson et al., 2019). NRF2 triggers the expression of a number of genes responsible for the sequestration of free iron and enzymes that neutralize the oxidative potential of ROS (Tonelli et al., 2018). Therefore, the accumulation of both internal and external lipids results in HIFlα stabilization and the adoption of AG. It seems that macrophages are predetermined for this detoxification function by exploiting a whole set of genes involved in lipid metabolism and thus help to cope with ectopic lipid deposition (Bobryshev et al., 2016).

Under conditions in which macrophages are exposed to excessive lipids for a time-restricted period, such as aerobic exercise, intermittent fasting, and caloric restriction, induction of mild mitochondrial stress may be beneficial for the organism. This phenomenon, called mitohormesls, alleviates systemic insulin signaling, which has a positive impact on lifespan (Ristow and Schmeisser, 2014). Nevertheless, prolonged exposure of macrophages to lipids leads to the adoption of pro-inflammatory phenotypes and chronic insulin resistance (Shin et al., 2017). During obesity, macrophages are thought to cause cytokine-induced insulin resistance in adipose tissue, the liver, and, subsequently, the whole organism (Marette, 2002; Tilg and Hotamisligil, 2006; Makki et al., 2013).

Activation of macrophages by excessive lipids may explain several metabolic syndromes such as adipose tissue inflammation, non-alcoholic liver steatosis, atherosclerosis, diabetes, and cachexia. This hypothesis is in concordance with clinical observations and experiments carried out on mice, in which the amelioration of macrophage polarization by anti-inflammatory agents and drugs affecting lipid metabolism leads to significant improvement of these syndromes in obese individuals (Bellucci et al., 2017; Koelwyn et al., 2018).

Although the term “aerobic glycolysis” *sensu stricto* refers to lactic acid fermentation of glucose, here we perceive it as a complex phagocyte metabolic program including, in addition, increased pentose phosphate pathway, lipid synthesis, and the mevalonate pathway, as well as a rewired flow of the Krebs cycle (Mills and O’Neill, 2016; Nonnenmacher and Hiller, 2018). Such metabolic adaptation affects also nutritional demands of these cells and makes them functionally dependent on external supplementation. Since the availability of nutrients may become limiting for the adoption of bactericidal polarization (Nagy and Haschemi, 2015; Ganeshan et al., 2019), they have to secure sufficient availability of sources in circulation and gain an advantage over the surrounding tissues in their use. Therefore, activated professional phagocytes release signaling factors regulating both local and systemic energy in order to usurp enough sources for an acute immune response (Khovidhunkit et al., 2004; Soeters and Soeters, 2012; Straub, 2014; Dolezal, 2015; Figure 1 and Box 1).

Besides the mobilization of sources from central energy-storing organs, such as adipose tissue and the liver, it is fundamental to limit the consumption of nutrients by other processes unrelated to the immune response (Almajwal et al., 2019). The privileged status of immune cells in reaching the nutrients is justified since making the immune response the most efficient is often a question of life and death. Although such behavior of the immune cells is for the sake of the individual, the usurpation of sources may be interpreted as selfish if viewed from the perspective of inter-organ competition for sources. Immune cell-derived signaling factors responsible for such systemic metabolic switch may be hence called selfish immune factors (SIFs) (Bajgar et al., 2015; Dolezal et al., 2019).

Insulin signaling is the central signaling pathway regulating the balance between anabolic and catabolic processes in the body (Schwartsburd, 2017). We may, therefore, presume that antagonism of insulin signaling is the most straightforward strategy to reroute energy flows from maintenance, growth, and reproduction to its fast utilization by the activated immune system. Cytokine-induced deterioration of insulin signaling leads to an increased titer of circulating energy-rich compounds such as glucose, lipoproteins, and amino acids (Felig et al., 1969; Salazar et al., 2018; Cho et al., 2019). The impact of infection-induced insulin insensitivity on the systemic metabolism highly resembles hyperglycemia and hyperlipidemia as hallmark states of chronic insulin resistance and cachexia (Khovidhunkit et al., 2004; de Luca and Olefsky, 2008; Shi et al., 2019). However, the regulation of energy homeostasis in mammals is substantially influenced also by other metabolism-related hormones such as cortisol and catecholamines, particularly noradrenalin and norepinephrine that should not be omitted for their effects on nutrient mobilization in situation of metabolic stress (Marik and Bellomo, 2013).

In the presented perspective, insulin resistance and subsequent pre-cachectic state induced by immune cell-derived factors may be perceived as an adaptive metabolic adjustment essential for the effective fight of invading pathogens. However, mobilization of nutrients and their altered distribution in the body may become detrimental if chronic and may progress to the development of several human pathological states.

In the following paragraphs, we would like to present several lines of evidence supporting this perspective. Although gained mostly by the research of immuno-metabolism in insects, these observations are in concordance with many data from mice models and humans. Since the metabolic switch of innate immune cells is best comprehended for macrophages, we will focus in this review mainly on these cells. The hypothetical model discussed in this review is based on knowledge of biology of both mammalian macrophages as well as *Drosophila* professional phagocytes, called plasmatocytes. Their basic characteristics and the features resembling mammalian macrophages and neutrophils are further described in Box 3. To specify that the presented information concerns *Drosophila* phagocytes, these cells will be always denoted here as plasmatocytes.

**BOX 3. *Drosophila* as a model for immuno-metabolic research.**

Over the last century, *Drosophila* has become a very universal and suitable model organism for the study of many human diseases. The simplicity of *Drosophila*, the existence of readily available transgenic strains, as well as the possibility of tissue-specific and time-limited knockdown of a particular gene make *Drosophila* one of the most suitable model organisms for the study of complex systemic metabolic syndromes (Duffy, 2002). In addition, approaches that previously could not be applied due to the lack of input material from such a small organism are now possible due to the greater sensitivity of analytical techniques in recent years (Cheng et al., 2018).

The *Drosophila* immune system consists of several layers of protection of an individual, which consist of two main branches of the humoral and cellular immune response. In addition to immune cells, the fat body also participates in immune responses, as the central metabolic organ supports the immune response by releasing resources and producing antimicrobial peptides (Melcarne et al., 2019). Although *Drosophila* may develop a characteristic immune response against underlying types of pathogens, such as gram-positive and gram-negative bacteria, viruses, and fungi, the adaptive immune response in *Drosophila* has not been reliably demonstrated (Ferrandon et al., 2007). The *Drosophila* and mammalian immune systems display a surprising level of homology in the major immune signaling pathways. The antibacterial response consists of the activation of the Toll and Imd signaling pathways, supported by the usual JNK and HIFlα stress response (De Gregorio et al., 2002).

**FIGURE 2:**
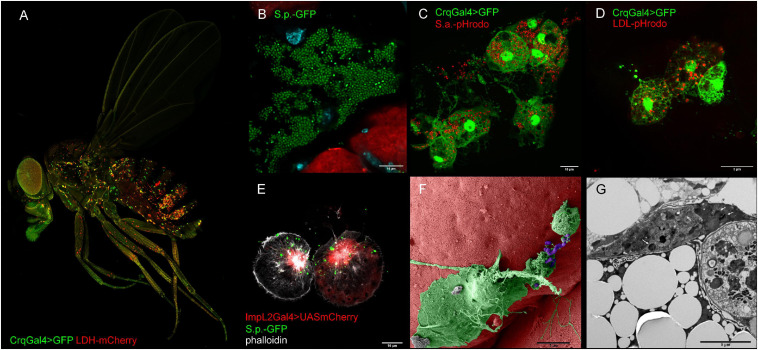
Representative confocal and electron microscopy images of *Drosophila* macropahges. **(A)** Adult *Drosophila* bearing a genetic construct that enables visualization of macrophages (in green HmlGal4 > UAS2xeGFP) and tissue expressing lactate dehydrogenase (LDH-mCherry). **(B)** Confocal image depicting growth of streptococcus in dissected *Drosophila* abdomen (green—*S. pneumoniae*, red pericardial cells, cyan—DAPI). **(C)** Confocal image depicting phagocytic events by injection of *Drosophila* adult with pHrodo^TM^ Red *S. aureus* Bioparticles^TM^ Conjugate. Macrophages are visualized by endogenously expressed GFP (Crq > GFP) (green—macrophages, red—phagolysosomes). **(D)** Confocal image depicting endocytosis of low-density lipoproteins by injection of adult fly with pHrodo^TM^ Red-LDL. Macrophages are visualized by endogenously expressed GFP (Crq > GFP) (green—macrophages, red—LDL-containing late endosomes). **(E)** ImpL2-expressing macrophages interacting with fluorescently labeled *S. pneumoniae* (green—*S. pneumoniae*, red—ImpL2 Gal4 > UAS mCherry, white—phalloidin). **(F)** Pseudo-colored scanning electron micrograph of a macrophage interacting with *S. pneumoniae* (green—macrophage, purple—*S. pneumoniae*). **(G)** Transmission electron micrograph of *S. pneumoniae* bacteria (white arrows) in a macrophage. Crq, croquemort; ImpL2, imaginal morphogenesis protein late 2; LDL, low-density lipoproteins; S.p., *Streptococcus pneumoniae*.

Some observations suggest that the innate immune response to invading pathogens shows certain features of trainability, but the mechanism of this process remains unclear. *Drosophila* immune cells, called hemocytes, include prohemocytes, plasmatocytes, crystall cells, and lamellocytes. While crystal cells and plasmatocytes are mainly involved in the encapsulation and melanization of foreign objects in the hemolymph, plasmatocytes represent a population of professional phagocytes (Melcarne et al., 2019; Figure 2).

Plasmatocytes are the most abundant population of cells in both larvae and adult flies (Figure 2A). These functionally versatile cells are involved in many biological processes (Figure 2F), from embryonic morphogenesis, metamorphosis, and wound healing to protection against invading pathogens (Banerjee et al., 2019). Because phagocytosis and bacterial killing are highly conserved at the level of cell biology, *Drosophila* plasmatocytes show an exceptional level of similarity to cells of the mammalian innate immune system, especially macrophages and neutrophils. Indeed, plasmatocytes use the same metabolic and signaling pathways for pathogen uptake and destruction in phagolysosomes (Figure 2C) as their mammalian counterparts, including the involvement of a plethora of homologous genes (Browne et al., 2013).

Although plasmatocytes are predominantly considered in the literature as a homogeneous population of phagocytic cells, a single cell transcriptomic analysis of the immune-stimulated larval hemocytes revealed a surprising level of their variability. However, the research of the plasmatocyte subpopulation is still at the beginning and far from distinguishing tissue-resident or specifically primed plasmatocyte subsets (Cattenoz et al., 2020; Tattikota et al., 2020).

Recently, the concept of immuno-metabolism has been developed in mammals, which indicates that several populations of mammalian immune cells must adopt a specific cellular metabolism in order to perform the desired function (Galván-peña and O’Neill, 2014). Although there are still some doubts about an analogous mechanism for *Drosophila* plasmatocytes, several publications and transcriptomic data document this ability (Krejčová et al., 2019; Cattenoz et al., 2020; Ramond et al., 2020; Tattikota et al., 2020). These observations are necessary not only for a comprehensive understanding of the antibacterial immune response but may become a base for research of many other human diseases that are connected with the pathological metabolic polarization of mammalian immune cells.

Despite the undeniable benefits of the *Drosophila* model for the study of human diseases, there are certain limits because many *Drosophila* organs and tissues show a lower level of complexity than in mammals.

*Drosophila* is currently used extensively to study insulin resistance. *Drosophila* and mammalian insulin signaling share major components at the level of cell biology (Álvarez-Rendón et al., 2018). However, certain significant differences also need to be taken into account. *Drosophila* carries eight insulin-like peptides (DILP1-8) that show structural homology to either mammalian insulin or relaxin. Analogous to mammals, *Drosophila* insulin signaling also reflects the current metabolic status of the individual. DILPs 2, 3, and 5 are thus released by specialized neurosecretory cells in the *Drosophila* CNS to regulate reproduction, growth and lifespan. While most DILPs activate a single *Drosophila* insulin receptor, DILP8 binds to its own LGR3 receptor. The situation in humans is even more complicated because, in addition to insulin, we can recognize two insulin-like growth factors, relaxin, as well as several insulin-like peptides. Insulin signaling activity is affected by many convergent signaling pathways and factors, such as hormones of a lipophilic nature, as well as insulin-binding proteins and IGFs (Nässel et al., 2015; Nässel and Broeck, 2016). Thus, an analogy can also be observed in the manner of insulin resistance induction.

Therefore, we believe that ongoing research on the role of the *Drosophila* immune system in the regulation of systemic metabolism will lead to new discoveries that can be generalized to human medical research.

We believe that we present here a compelling set of information to change the general conception of insulin resistance and pre-cachexia as clearly pathological states. This may help to better comprehend medical treatment in many human diseases.

## Macrophage Adoption of a Bactericidal Phenotype Is Nutritionally Demanding

Macrophages, as highly versatile cells, fulfill various tasks in the organism. Besides representing the front line of protection against invading pathogens, macrophages also clear apoptotic cellular debris, maintain tissue homeostasis, and participate in the formation of many morphological structures during development (Wynn et al., 2013; Gordon and Martinez-Pomares, 2017; Theret et al., 2019).

Not surprisingly, the various macrophage tasks require specific settings of cellular metabolism to obtain the optimal amount of metabolites and precursors required for the desired function. That may be depicted, for instance, in the metabolism of amino acid arginine. While macrophages participating in wound healing metabolize arginine to generate growth-promoting ornithine essential for wound reconstruction, bactericidal macrophages use the same amino acid as a precursor for the production of nitric oxide later applied as an efficient bactericidal agent (Mills et al., 2015). This revelation led to later identification of the full spectrum of macrophage polarization states characterized by their metabolic program, with the extremes represented by healing and bactericidal polarizations (Mosser and Edwards, 2008). Interestingly, the metabolic settings are determinative of macrophage function, and a mere metabolic setting has the potential to change the polarization phenotype (Galván-peña and O’Neill, 2014).

Upon pathogen infiltration, macrophages have to recognize, entrap, engulf, and destroy the invaders in the phagolysosome (Diskin and Pålsson-McDermott, 2018). There is no doubt that these processes are connected with excessive energy expenditure and a need for a synthesis of a high amount of precursors for the production of bactericidal agents, signaling molecules, as well as remodeling of cytoskeleton and cellular membrane. It has been estimated that the cellular membrane of activated macrophage turns over completely every 30 min due to accelerated endocytosis and micropinocytosis (Werb and Cohn, 1972). Besides membrane remodeling, phagocytosis also requires a high amount of energy. The ATP required for phagocytosis of a single polystyrene particle has been estimated to cost about 10^9^ ATP molecules (Karnovsky, 1962). The subsequent generation of a sufficient amount of ROS and myeloperoxidase for bacterial killing in the phagolysosome is another metabolically demanding process. The production of ROS, as well as compensation of its cytotoxicity, depends on sufficient availability of NADPH in cells. Therefore, macrophages must substantially increase the flow rate of the metabolic pathways producing this reducing agent (Panday et al., 2015).

**FIGURE 3:**
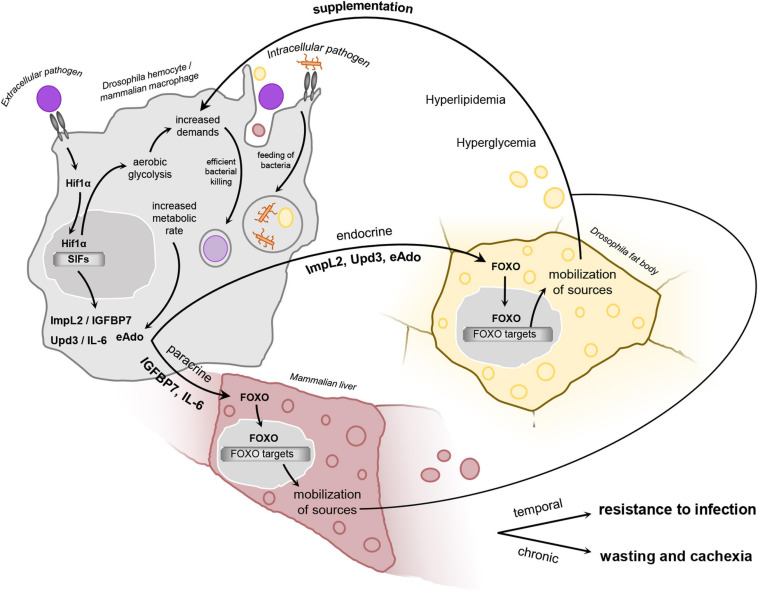
Schematic representation of the proposed hypothetical model. In infection-activated macrophages, HIF1α stabilization leads to adoption of aerobic glycolysis, which is a highly energy demanding metabolic program. Aerobic glycolysis is interconnected with the production of selfish immune factors. These molecules affect remotely the metabolism of the main storage organs via induction of insulin resistance, leading to FOXO nuclear translocation and induction of mobilization of sources. This results in elevated titer of circulating carbohydrates and lipids, which are thus utilized by bactericidal macrophages to supplement their increased energy demands. Such inter-organ communication is essential for resistance to infection by extracellular pathogen, but may be maladaptive upon its chronic activation or in case of infection by intracellular bacteria. Hif1α, hypoxia-inducible factor 1 α; FOXO, forkhead box O; Upd3, unpaired 3; ImpL2, Imaginal morphogenesis protein late 2; IGFBP7, insulin-growth factor binding protein 7; IL-6, interleukin 6; eAdo, extracellular adenosine; SIFs, selfish immune factors.

To cover the sudden requirements arising from bactericidal function, macrophage has to adjust the overall metabolic setup, i.e., glycolysis, pentose phosphate pathway, mevalonate pathway, as well as the mitochondrial cycle of tricarboxylic acids and oxidative phosphorylation (Galván-peña and O’Neill, 2014). Such a complex rearrangement is orchestrated by central metabolic regulator Hypoxia-inducible factor 1 alpha—*Hif1α* (Corcoran and O’Neill, 2016; Wang et al., 2017). This stress-related transcription factor, originally discovered in research of hypoxia, is constitutively produced and degraded by all cells in the body (Marxsen et al., 2004). That is particularly important for immediate initiation of Hif1α activity since mere inhibition of its degradation suffices to stimulate expression of its target genes (Watts and Walmsley, 2019). Stabilized HIF1α triggers the expression of more than a hundred genes under the control of the hypoxia response element (Dengler et al., 2014). The unique metabolic program established by the activity of HIF1α is generally called aerobic glycolysis. Between HIF1α-target genes, we can find mostly enzymes directly participating in metabolic pathways upregulated in aerobic glycolysis or regulating their flow rate, as will be mentioned below (Figure 3).

Many different signaling cascades converge on prolyl hydroxylase dehydrogenase (PHD), the enzyme responsible for HIF1α degradation. PHD requires several metabolic products as essential cofactors for its enzymatic activity. From the most prominent, we should mention oxygen, Fe^2+^ ions, and α-ketoglutarate as a product of canonically running Krebs cycle (Iommarini et al., 2017). Although originally described in hypoxia, HIF1α stabilization may be achieved even under normoxic conditions as may be observed in macrophages stimulated by pathogen-associated molecular patterns or pro-inflammatory cytokines (Iommarini et al., 2017). These ligands activate toll-like receptor 4 (TLR4), which further enhances a Nuclear Factor kappa B (NF-κB)-signaling pathway. As an outcome of NFkB activity, the cytosolic Fe2^+^ ions are sequestered by the major iron-storage protein ferritin. Lack of Fe^2+^ as a crucial cofactor of PHD thus causes HIF1α stabilization and substantial remodeling of overall cellular metabolism (Siegert et al., 2015). It should be noted that TLR4 may also be activated by endogenous ligands such as extracellular matrix components, oxidized lipids, and lipoproteins (Erridge, 2010).

Besides extracellular stimuli, HIF1α stabilization may be achieved by the cytosolic accumulation of several metabolic intermediates originating from the mitochondrial Krebs cycle. It has been documented that lactate, succinate, itaconate, pyruvate, and 2-hydroxyglutarate impair PHD ability to degrade HIF1α (Koivunen et al., 2007; Bailey and Nathan, 2018). This interconnection seems to be adaptive for overcoming hypoxic states since an accumulation of these metabolites in the cytosol is a hallmark of mitochondrial dysfunction (Garedew and Moncada, 2008; Prabakaran, 2015). Even though this mechanism enables cells to reflect their current metabolic state, it makes HIF1α stabilization dependent on elevated flow through metabolic pathways enhanced in aerobic glycolysis. Therefore, cells employing aerobic glycolysis are highly sensitive to the availability of sources. Early experiments using LPS as a classic way to activate macrophages showed that macrophages functionally depend on sufficient concentrations of glucose, glutamine, and lipids in the culture medium (Newsholme et al., 1986). Further *in vitro* investigation of nutrient uptake and trafficking fully elucidated the complexity of HIF1a-mediated metabolic changes and the utilization of these nutrients by bactericidal macrophages (Stunault et al., 2018).

An immense uptake of glucose is one of the hallmarks of bactericidal macrophages. That may be explained by its utilization as a primary energy resource as well as a substrate for the generation of NADPH and nucleotides in the pentose phosphate pathway (Yamashita et al., 2014). Consistently, *glucose-6-phosphate dehydrogenase*, which catalyzes the first step in pentose phosphate pathway, is known to be triggered by HIF1α (Gao et al., 2004). Interestingly, the glucose energy potential is not fully exploited since pyruvate as the end-product of glycolysis is not entering the mitochondria for its full oxidation. Due to HIF1α transcriptional activity, it is instead preferentially converted to lactate by *lactate dehydrogenase* and excluded from the cell through *monocarboxylate transporter 4* (Firth et al., 1995; Kim et al., 2006). Via increased glycolytic flux, cells avoid the time-consuming transport of pyruvate into the mitochondrial lumen that otherwise represents the rate-limiting step of ATP generation (Thomas and Halestrap, 1981). Thanks to that, the glycolytic flux may be increased even a hundred times, thus fully compensating for the lower efficiency of ATP generation. The acceleration of glycolysis is also under the control of HIF1α, which regulates the expression of rate-limiting glycolytic enzymes *hexokinase II* and *phosphofructokinase-1* (Riddle et al., 2000; Obach et al., 2004). Pyruvate conversion into lactate, together with pentose phosphate pathway, serves as a mechanism generating sufficient amounts of NADPH to be utilized for ROS production as well as self-protection against its detrimental effects (Riganti et al., 2012).

Despite the generation of sufficient amounts of ATP by glycolysis, mitochondrial metabolism is still crucial for activated macrophages (Sancho et al., 2017). Indeed, many Krebs cycle intermediates have been shown to be essential for macrophage bactericidal function. Since HIF1α-elevated expression of *pyruvate dehydrogenase kinase* diverts the pyruvate from entering the mitochondria, there must be an alternative way for Krebs cycle supplementation. The flow of the Krebs cycle is sustained by using glutamate as an initial precursor for the synthesis of Krebs cycle intermediates. To supplement the Krebs cycle by glutamate, HIF1α increases the expression of glutamine transporters SLC1A5 and SLC38A2 (Chen et al., 2001). Since the Krebs cycle is replenished from a different direction than usual, it produces several intermediates in opposite directions and was therefore referred to as the “broken Krebs cycle” (O’Neill, 2015). Consequently, the concentration of several Krebs cycle intermediates varies substantially in the cytosol. While overproduced itaconate and fumarate are used directly to fight the pathogen extracellularly, citrate is used as a substrate for the synthesis of fatty acids and glutathione (Rouzer et al., 1982). However, the broken Krebs cycle does not generate enough precursors to fuel the oxidative phosphorylation. The canonical function of oxidative phosphorylation is thus disabled, and cells cannot employ cellular respiration (Ramond et al., 2019). Although bactericidal macrophages generate ATP independently from oxygen, their activity is often associated with a high oxygen consumption rate (OCR) when metabolically analyzed under controlled *in vitro* conditions (Van den Bossche et al., 2015). This can be explained by the massive utilization of oxygen for the generation of reactive oxygen and nitric species (ROS/RNS) later used for bacterial killing in phagolysosomes and oxidative burst (Forman and Torres, 2002). Indeed, an expressional increase in *Nitric oxide synthase* is triggered by the transcriptional activity of HIF1α (Matrone et al., 2004). ROS are produced by the NADPH-oxidase complex as well as the reversed mitochondrial electron transport chain. Production of ROS/RNS thus depends on the utilization of ATP, NADPH, and their effective regeneration (Xu et al., 2016; Scialò et al., 2017).

A considerable amount of ROS must be generated for bacterial killing in phagolysosomes. However, with increasing concentration of ROS, also the risk of lipid peroxidation and subsequent cell death rises. Bactericidal macrophages invest many sources to cascades producing a sufficient amount of neutralizing reductive compounds. Citrate and glutamate are exploited for the generation of glutathione, which protects thus macrophages from self-harming by otherwise bactericidal ROS (Kwon et al., 2019).

Finally, yet importantly, the difference can also be seen in the utilization of lipids if comparing quiescent and bactericidal macrophages. While resting macrophages use a relatively small amount of lipids mainly as a source of energy from fatty acid oxidation, upon infection, HIF1α-induced activity of sterol regulatory element-binding proteins and peroxisome proliferator-activated receptors lead to the accumulation of fatty acids and cholesterol (Shen and Li, 2017; Mylonis et al., 2019). That may be attributed to increased uptake of lipids in the form of lipoproteins as well as a rise in lipid synthesis. Uptake of lipoproteins [via scavenger receptor CD36, very-low-density lipoprotein receptor (VLDL-R) and low-density lipoprotein receptor-related protein 1 (LRP1)] as well as their synthesis increases in a HIF1α-dependent manner (Krishnan et al., 2009; Castellano et al., 2011; Mylonis et al., 2012; Shen et al., 2012; Maier et al., 2017), which further supports the perception of HIF1α as a master-regulator of aerobic glycolysis in bactericidal macrophages. However, the involvement of this regulation upon infection has not been fully comprehended yet. Contrary, the utilization of fatty acids for energy generation via fatty acid oxidation is significantly decreased upon HIF1α stabilization (Remmerie and Scott, 2018). Even though the use of lipids by macrophages upon infection has not been fully elucidated yet, we can presume their deployment for remodeling of the cellular membrane, formation of cholesterol rafts, synthesis of catecholamines, trained immunity, as well as inflammasome activation (Bekkering et al., 2018; Remmerie and Scott, 2018).

As we depicted above, the adjustment of macrophage central metabolic pathways is fundamental for the engulfment of bacteria and its killing. However, this relationship has been omitted for a long time in insects. Nevertheless, phagocytosis and clearance of invading pathogens is an evolutionarily highly conserved process even on the molecular level and, therefore, plasmatocytes as *Drosophila* professional phagocytes (Figure 2 and Box 3) should have the same requirements for energy and precursors (Stuart and Ezekowitz, 2008; Browne et al., 2013). The position of plasmatocytes in fly’s body (Figure 2A), their morphology (Figures 2C–G), as well as their ability to phagocytose bacteria (Figures 2C,E–G and Box 3) and uptake LDLs (Figure 2D) are depicted in Figure 2.

Thus, we can hypothesize that basically, all professional phagocytes performing bactericidal function should undergo the switch toward aerobic glycolysis upon their activation. This notion is supported by observations made by Anderson and his colleagues, who investigated the metabolic demands of cockroach hemocytes during phagocytosis *in vitro*. They revealed that insect hemocytes are functionally dependent on uptake of glucose, glutamine, and lipids from cultivation media (Anderson et al., 1973; Ratcliffe and Rowley, 1975). That may be supported by transcriptomic data characterizing *Drosophila* immune cells with various stimuli. In larvae, both differentiating and proliferating immune cells display hallmarks of increased glycolytic rate and conversion of pyruvate to lactate resembling aerobic glycolysis (Irving et al., 2005; Johansson et al., 2005; Bajgar et al., 2015; Ramond et al., 2020). The versatility of *Drosophila* immune cells and their metabolic response to the activating stimuli may be further documented by the single-cell transcriptomic analysis published recently (Tattikota et al., 2020), which shows the above-mentioned patterns in raw data. According to these data, larval hemocytes display increased expression levels of lipid-scavenging receptors and genes for import and metabolism of lipids in the Krebs cycle. Moreover, a subpopulation of immune cells bearing lamellocyte markers displays metabolic shift toward aerobic glycolysis upon wasp infestation.

It has been proven experimentally that even adult fly plasmatocytes perform the switch to aerobic glycolysis upon streptococcal infection *in vivo* (Krejčová et al., 2019). In analogy to their mammalian counterparts, *Drosophila* plasmatocytes require the activity of HIF1α for induction of aerobic glycolysis and, in response to infection, display substantially increased glucose and lipid uptake (Krejčová et al., 2019, 2020). In concordance with that, the rate of glycolysis, as well as the production of lactate, is increased in these cells. However, the complex metabolic characterization concerning particularly mitochondrial metabolism still remains to be fully explored. In this experimental setup, plasmatocyte function is central for limiting the bacterial burden during the first 24 h post-infection. Decreased efficiency of phagocytosis and bacterial killing leads to the death of the individuals. Interestingly, the cellular metabolic switch is accompanied by an adjustment of the systemic metabolism of flies when both adoption of aerobic glycolysis by plasmatocytes and induction of hyperglycemia and hyperlipidemia are essential for resistance during the acute phase of the infection. Since adoption of aerobic glycolysis by plasmatocytes is epistatic to adjustment of systemic metabolism, we may anticipate the existence of signaling factors mediating this interorgan crosstalk (Bajgar and Dolezal, 2018; Krejčová et al., 2019, 2020; Figure 1 and Box 1).

In conclusion, the adoption of aerobic glycolysis as a metabolic program fundamental for effective bactericidal function results in increased demands for external sources. Since these sources may be depleted rapidly in the local microenvironment (Kedia-Mehta and Finlay, 2019), we suggest that one of the possible ways how to ensure resource supplementation is the release of immune cell-derived signaling factors to affect systemic metabolism (Figure 1 and Box 1). The character of these signaling factors will be considered in the following paragraphs.

## Adoption of Aerobic Glycolysis Is Connected With the Release of Systemic Signaling Factors

As described in the previous paragraphs, macrophage activation is connected with enhanced nutritional demands due to the adoption of aerobic glycolysis and a high activity of these cells. Macrophages are expected to release signaling factors to usurp enough sources from other non-immune organs and tissues. Thus, the immune response becomes a privileged physiological process above other processes in the body. However, redistribution of sources may be limiting for concurrent physiological processes based mainly on anabolic metabolism (Ganeshan et al., 2019; Kedia-Mehta and Finlay, 2019). From the perspective of inter-organ signaling, the immune system behaves selfishly in competition for energy sources and releases SIFs that mediate this signaling (Figure 1 and Box 1). Based on the knowledge of insect SIFs, we may propound several hypothetical features to be met by these factors. This approach may help to identify possible novel SIFs in mammals.

Firstly, we expect the SIFs to be released by activated immune cells as a reflection of their nutritional status and adoption of HIF1α-driven aerobic glycolysis. There are two ways to translate the information about the increased demands linked to the adoption of aerobic glycolysis into the production of SIFs. SIF production may be a part of the transcriptional program associated with the metabolic switch directed by either HIF1α or other transcriptional factors involved in immune cell polarization—for example, JNK and NfKB. Thus, the remodeling of cellular metabolism of these cells and concurrent production of SIFs may be intimately interlinked. Alternatively, certain metabolites, generated as a product of some highly active metabolic pathways in aerobic glycolysis, may serve as potential SIFs as well (Figure 3).

Whether or not SIFs are linked to a transcriptional program or to the metabolic status of the cells, they should be released during the early phase of the acute immune response. Although it has been shown that macrophages are endowed with certain nutritional stores, they barely suffice for the initial few hours of their activation (Ma et al., 2020). This fact has been documented by many clinical data as well as experimental studies describing the progress of infection (Imran and Smith, 2007; Scott et al., 2019). Last, but not least, we should consider the potential of SIFs to spread through the body and affect systemic nutrient expenditure.

Assuming that the nutritional requirements of activated immune cells are the primary motivation for SIF release, we can look for a parallel in neoplastic tumors and hypoxic tissues, because they all use HIF1α-driven aerobic glycolysis (Escoll and Buchrieser, 2018; Miska et al., 2019; Box 4). Based on that presumption, we may preselect several cancer-derived cachectic factors that also occur in hypoxia. In the following paragraphs, we will address three immune signaling factors that meet the above criteria and represent the potential SIFs in *Drosophila* [extracellular adenosine (eAdo), insulin/IGF antagonist Imaginal morphogenesis protein late 2 (ImpL2), and cytokine Unpaired3 (Upd3)] (Figure 3).

**BOX 4. Cancer and bactericidal macrophages display a similar cellular metabolic setup.**

It is almost 100 years since the discovery that cancer cells preferentially employ glucose fermentation as an oxygen-independent source of ATP even when sufficiently supplied with oxygen (Warburg et al., 1927; Warburg, 1956). This metabolism was thought to be unique for cancer cells and was called the Warburg effect, named after its discoverer. Since the adoption of the Warburg effect yields eighteen times less ATP generated from one molecule of glucose compared to oxidative phosphorylation, the benefits arising from the use of such a metabolic program appeared unlikely. The adoption of the Warburg effect was thus attributed to disturbed mitochondrial function. However, this explanation cannot elucidate the similar observations made in yeasts that often use anaerobic metabolism despite the constant level of oxygen in the culture. This phenomenon is known as the Crabtree effect, which suggests an adaptive significance for such metabolic settings (de Deken, 1966; Diaz-Ruiz et al., 2011). Later research has shown that this mechanism is also utilized by other highly active or dividing cells, such as embryonic stem cells and activated bactericidal macrophages, and the term aerobic glycolysis has been introduced for this metabolic adaptation (Jones and Bianchi, 2015). This motivated scientists to find an explanation for why cells in certain situations prefer to switch to this metabolic regime and what the benefits are.

Using modern metabolomics techniques, it has been found that the lower yield of ATP is compensated by the increased glycolytic rate and that this metabolic setting represents an advantage in the production of essential precursors promoting cell growth, division, and active participation in many biological processes (Burns and Manda, 2017). As a result, these cells are dependent on an increased supply of nutrients. It is now clear that neoplastic cancer cells alter all major cellular metabolic pathways and that there is a high similarity in metabolism between cancer and bactericidal macrophages (Escoll and Buchrieser, 2018). It is generally accepted that neoplastic cancer cells represent a significant energy burden for patients compared to benign tumors of the same size. The malignancy of these tumors depends on the induction of systemic metabolic changes such as insulin resistance and cachexia (Nagao et al., 2019).

Interestingly, the pro-cachectic effect of tumors is interconnected with the adoption of HIFlα -dependent aerobic glycolysis (Koltai, 2020). It has been outlined that cancer may be perceived as a metabolic syndrome comprising cancer-induced insulin resistance and cachexia as mechanisms to usurp enough nutrition from the host’s anabolic processes to support tumor growth and metastatic spreading (Porporato, 2016). In concordance with that, cachexia is thought to cause about 20% of deaths in cancer patients and accompany up to 80% of advanced cancer states (Fonseca et al., 2020). Besides metabolic profile, cancer cells also share with bactericidal macrophages the production of several pro-inflammatory cytokines with impact on systemic metabolism (Liou, 2017). Therefore, research on these factors and their involvement in the induction of insulin resistance and cachexia upon infection should be considered.

Adenosine is a purine metabolite naturally occurring at low concentrations in all living cells. Nevertheless, its concentration may rise substantially as a reflection of increased activation of cellular metabolism (Eltzschig, 2013). Adenosine is formed in the cells as an outcome of the enormous consumption of ATP, the increased number of methylation events, as well as generation of reductive potential (Ham and Evans, 2012; Tehlivets et al., 2013; Sarkar et al., 2020). Accumulation of intracellular adenosine serves as a negative feedback signal on cellular metabolism via AMPK activation leading to quiescence (Aymerich et al., 2006). That is contradictory to the desired tasks of an activated immune system, and immune cells thus must expel excessive adenosine extracellularly (Sag et al., 2008). Since the quantification of intracellular adenosine is technically challenging under natural physiological conditions, its production by immune cells has to be presumed from indirect evidence. Nonetheless, the processes leading to the generation of intracellular adenosine are accelerated in activated macrophages employing aerobic glycolysis (Leonard et al., 1978; Vijayan et al., 2019; Silva et al., 2020). Aside from the intracellular source of adenosine, we should not omit its generation in an extracellular space, where it may be produced by ectonucleotidases bound to the surface of the immune cells (Zanin et al., 2012). Characteristic producers of adenosine in mammals are hypoxic endothelial and smooth muscle cells, activated immune cells, as well as cancerous tissues (Grenz et al., 2011; Silva-Vilches et al., 2018; Boison and Yegutkin, 2019). Recently, it has been shown that intracellular adenosine may be released by cultured human macrophages infected by *Leishmania* (Hsu et al., 2012). It is in concordance with an observation made in *Drosophila*, in which activated immune cells release adenosine via equilibrative nucleoside transporters upon an infestation of larvae by parasitoid wasps (Bajgar et al., 2015). Extracellular production of adenosine has also been described for murine macrophages upon their classic activation by LPS (Zanin et al., 2012). Although local rise in adenosine concentration has rather anti-inflammatory effects in mammals (Haskó and Cronstein, 2013), its systemic spreading may support immune response by mobilizing required energy substrates (Tadaishi et al., 2018). As an outcome of paracrine and systemic adenosine effects, we may observe overall metabolic suppression in the organism inducing thus, e.g., fatigue or hibernation (Davis et al., 2003; Olson et al., 2013). That is analogous to the observation made in infected *Drosophila* where adenosine directs mobilization of carbohydrates from adipose tissue and concurrently limits glucose consumption by other than immune tissues (Bajgar et al., 2015; Bajgar and Dolezal, 2018). Although the release of adenosine has not yet been experimentally linked to the adoption of aerobic glycolysis in activated immune cells, it is well established that many genes involved in adenosine signaling are HIF1α targets (Bowser et al., 2017). Thus, we hypothesize that adenosine production may be directly linked to the adoption of aerobic glycolysis. This is in concordance with the observation of eAdo release from cancer cells, hypoxic tissues, as well as activated immune system (Schrader et al., 1977; Alam et al., 2015; Bajgar and Dolezal, 2018; Arab and Hadjati, 2019).

The second SIF—ImpL2—has been identified as a *Drosophila* cancer-derived cachectic factor (Kwon et al., 2015). This putative functional homolog of mammalian *insulin-like growth factor-binding protein 7* (IGFBP7) is known to be released from experimentally induced cancer cells in adult flies. ImpL2 affects the metabolism of adipose tissue via insulin resistance and induces the mobilization of nutrients subsequently exploited by the tumor for its own growth (Kwon et al., 2015; Figueroa-Clarevega and Bilder, 2015). ImpL2 is documented to be released from tumors, which growth was induced either by loss of cell polarity or overexpression of transcription coactivator *Yorkie* (Bunker et al., 2015; Kwon et al., 2015). Importantly, these tumors are known to rely metabolically on aerobic glycolysis (Wang et al., 2016).

A remarkable release of ImpL2 was also observed from tissues undergoing experimentally-induced hypoxia and mitohormesis, where its expression reflected the mitochondrial dysfunction (Allee, 2011; Owusu-Ansah et al., 2013). The link between HIF1α and ImpL2 production has been revealed by comparing ImpL2 transcript abundance in response to hypoxia for wild-type and HIF1α homozygous mutant adult flies. Moreover, experimentally increased HIF1α expression is sufficient for enhanced ImpL2 protein levels (Allee, 2011). The role of HIF1α in the regulation of ImpL2 production has been suggested for infection-activated plasmatocytes (Krejčová et al., 2020). It has been revealed that the rise in ImpL2 expression in plasmatocytes (Figure 2E) is dependent on HIF1α activity in these cells upon infection. Thus, HIF1α directs not only the metabolic switch to aerobic glycolysis but also ImpL2 expression. That is further supported by the occurrence of four hypoxia response elements in the regulatory sequence of the ImpL2 genomic region. We thus may claim that bactericidal plasmatocytes produce ImpL2 as a reflection of HIF1α-driven aerobic glycolysis (Krejčová et al., 2020). Interestingly, plasmatocytes produce ImpL2 not only in response to the recognition of invading pathogens but also in response to their exposure to excessive lipids, as it has been documented for high-fat-diet fed flies (Morgantini et al., 2019). Since ImpL2 is known to bind *Drosophila* insulin-like peptides, its effects on systemic metabolism can be accounted to the abrogation of insulin signaling (Honegger et al., 2008).

The last SIF discussed here is a *Drosophila* cytokine Upd3. Based on its structural and functional similarities, it is considered to be a functional homolog of mammalian cytokine IL6 (Oldefest et al., 2013). In analogy to its mammalian counterpart, Upd3 also acts as a ligand for the JAK-STAT signaling pathway. Upd3 production is crucial in the regulation of many physiological processes, ranging from embryogenesis and larval growth and development to stress response, such as in tissue damage, loss of cell polarity, metabolic stress, and bacterial infection (Jiang et al., 2009; Wang et al., 2014; Woodcock et al., 2015). Under such situations, Upd3 production is triggered by the activation of JNK by loss of cell polarity, recognition of bacterial pathogens, or increased accumulation of ROS (Jiang et al., 2009). Immune cells are one of the prominent producers of Upd3 in adult flies. In response to tissue damage, bacterial infection, or exposure to oxidized lipids, Upd3 expression rises in these cells substantially (Agaisse et al., 2003; Woodcock et al., 2015; Chakrabarti et al., 2016; Shin et al., 2020). Systemic Upd3 subsequently triggers JAK-STAT signaling in non-immune tissues and activates a stress response primarily in the gut and the fat body. While in the gut, Upd3 induces regenerative proliferation and maintenance of integrity, in the fat body, it induces a Foxo-driven transcriptomic program, leading to a mobilization of lipid stores (Chakrabarti et al., 2016; Shin et al., 2020).

Interestingly, Upd3 production is induced under a similar condition to ImpL2. Indeed, both are produced from cancer and hypoxic cells as well as from plasmatocytes responding to bacterial infection, excessive lipids, or tissue damage (Agaisse et al., 2003; Bunker et al., 2015; Shin et al., 2020). The interconnection of Upd3 production with HIF1α transcriptional activity has been observed for hypoxia-responsive neurons in the central nervous system of *Drosophila* larvae. Upd3 released by these cells has a remote impact on insulin signaling in adipose tissue and, thus, supports the proliferation of immune cell progenitors in lymph glands (Cho et al., 2018).

From the above-mentioned, we may suggest that Upd3 production reflects a situation of cellular metabolic stress. However, the direct link between plasmatocyte aerobic glycolysis and Upd3 production has not been satisfactorily studied to date. A systemic effect of Upd3 may be attributed to the activation of a JAK-STAT cascade, which often leads to an alleviation of the insulin signaling pathway in target tissues (Yang et al., 2015; Kierdorf et al., 2020; Shin et al., 2020).

We propose that all three SIFs discussed here are produced by bactericidal immune cells due to their increased metabolic activity and the adoption of HIF1α-driven aerobic glycolysis. It is particularly interesting that the informing of metabolic demands is mediated by multiple factors involving the body’s central metabolic organs. However, it seems that their cooperative action ensures the supplementation of the immune system with sources (Figure 3).

## Immune Cell-Derived Factors Induce Mobilization and Targeted Delivery of Nutrients

The task of SIFs is to ensure sufficient supplementation of their producers with energy resources and nutrients necessary for their function.

The mechanism of resource redistribution consists of two parallel processes, the mobilization of resources from reserves and their subsequent delivery to the activated immune system. The energy suddenly required for protection against pathogen attack is usurped from anabolic processes such as the building of reserves, maintenance, growth, and reproduction. Therefore, SIFs are expected to mobilize the nutrients from central energy-storing organs and concurrently minimize their consumption by other immune response-unrelated tissues.

Since most physiological processes based on anabolism depend on the insulin signaling pathway (Schwartsburd, 2017), we can assume that the transition between insulin sensitivity and resistance may represent such a mechanism. We hypothesize here that ImpL2, Upd3, and adenosine represent examples of possible SIFs. Therefore, their impact on systemic metabolism with emphasis on the induction of insulin resistance will be considered in the following paragraphs.

Recently, it has been deciphered that ImpL2 is released from infection-activated plasmatocytes during acute immune response in *Drosophila* (Krejčová et al., 2020; Figure 3). However, a recently published RNA-Seq analysis of *Drosophila* larval plasmatocytes revealed neither an increase in ImpL2 transcripts upon septic injury nor enriched expression of ImpL2 in plasmatocytes (Ramond et al., 2020). That is in concordance with other observations showing that larval ImpL2 is expressed in the fat body rather than in circulating immune cells. That suggests a different role of ImpL2 in larva and adult immune system since, in adult flies, the subpopulation of plasmatocytes clearly displays a strong ImpL2 expression level, particularly of ImpL2 RA isoform (Krejčová et al., 2020). Interestingly, another single-cell analysis displays a clear subpopulation of larval plasmatocytes denoted according to a high level of ImpL2 expression as ImpL2-positive (Cattenoz et al., 2020).

Krejčová shows that ImpL2 subsequently affects the mobilization of carbohydrates and lipoproteins from the fat body, which results in their increased titer in circulation and their subsequent utilization by activated plasmatocytes (Figure 3). Several independent approaches document its impact on nutrient mobilization. It was shown that ImpL2 induces morphological changes in the fat body of infected individuals. The adipocytes display a significantly reduced amount of lipid stores, which are dispersed in the cytoplasm in an increased number of smaller lipid droplets. It is believed that the reduced diameter of the lipid droplets is advantageous for cells undergoing increased lipolysis since it makes the triglycerides more accessible to lipases located on their surface (Kühnlein, 2012). That is in concordance with the induction of Forkhead Box O (Foxo)-driven transcriptomic program, which triggers the expression of enzymes responsible for lipolysis and assembly and release of lipoproteins (Figure 3). Lipid mobilization in the form of lipoproteins is further supported by the change of relative representation of individual lipid classes in the fat body on behalf of phospholipids. Interestingly, a mere overexpression of ImpL2 in plasmatocytes is able to mimic the effects of infection in the fat body (Krejčová et al., 2020).

Foxo is known to regulate adipocyte metabolism upon metabolic stress conditions such as starvation, hypoxia, and elicitation of immune response. It has been reported that when starving or eliciting an immune response, Foxo is triggered by immune signaling cascades such as NF-κB, Toll, and IMD in the fat body (Molaei et al., 2019; Texada et al., 2019). Nevertheless, adipocyte insulin signaling has the power to counteract this nutrient-deliberating mechanism completely (Lee and Dong, 2017). Therefore, it is central for the organism to alleviate insulin signaling in these cells to induce mobilization of stores. ImpL2 is a perfect candidate for this role since it is known for its high affinity to *Drosophila insulin-like peptides* as well as experimentally administered human insulin (Honegger et al., 2008). Although the production of ImpL2 by plasmatocytes appears to be sufficient to induce changes in lipid metabolism of adipose tissue upon infection, another plasmatocyte-derived factor, Upd3, surprisingly targets the same signaling pathway in this organ (Krejčová et al., 2020; Shin et al., 2020).

There is a striking similarity between the effects accounted for ImpL2 and Upd3. Contrary to ImpL2, Upd3 affects the FOXO nuclear translocation via activation of the JAK/STAT signaling pathway in the fat body and induces insulin resistance in adipocytes downstream of insulin receptor (Shin et al., 2020). That may be accomplished via affecting the phosphorylation status of effector kinase AKT. Interestingly, also Upd3 itself can induce mobilization of lipid stores into the circulation (Woodcock et al., 2015). Redundancy of ImpL2 and Upd3 effects suggests that it is adaptive to inhibit insulin signaling in adipose tissue by multiple SIFs to secure mobilization of sources upon infection. Also eAdo affects adipose tissue metabolism in response to infection in *Drosophila*. While the effects of ImpL2 and Upd3 are manifested mainly by the mobilization of lipid stores, eAdo affects the level of expression of glycogen metabolizing enzymes through its receptor. eAdo induces hyperglycemia upon infection via depletion of adipose tissue glycogen stores (Bajgar and Dolezal, 2018). However, its effect on lipid metabolism has not been sufficiently investigated yet.

We may conclude that immune cell-derived SIFs induce adipocyte insulin resistance leading to mobilization of sources from adipose tissue and their utilization by activated immune cells (Figure 3).

Besides mobilization of sources, SIFs also often silence the nutrient consumption of tissues that are not involved in the immune response. Interestingly, all the SIFs discussed here are known to silence anabolic processes in these tissues in certain situations. Production of one factor by macrophages thus regulates concurrently both mobilization of sources and suppression of physiological processes competing with the immune response for resources.

The effect of ImpL2 on anabolic processes has been observed during the fly’s development and upon experimental induction of cancer. An increased titer of circulating ImpL2 alleviated insulin signaling and thus decreased metabolic muscle rate and caused fragmentation of muscle mitochondria (Figueroa-Clarevega and Bilder, 2015; Kwon et al., 2015; Lee et al., 2018). In addition, these individuals displayed disrupted ovary maturation and mobilization of sources leading to wasting-induced cachexia (Figueroa-Clarevega and Bilder, 2015; Kwon et al., 2015). We can hypothesize that plasmatocyte-derived ImpL2 may have similar effects upon infection, although not with as significant phenotypes as in cancer because upregulation of the ImpL2 gene in these experimental systems resulted in concentrations far beyond those occurring naturally.

Also, the effects of Upd3 on muscle metabolism have been investigated. Plasmatocyte-derived Upd3 has been shown to limit remote lipid accumulation in muscles to maintain lipid homeostasis in the tissue via alleviation of insulin signaling in these cells through activation of the JAK-STAT singling pathway, which is documented by decreased pAKT occurrence (Kierdorf et al., 2020). We suggest that such a mechanism may also be involved in the regulation of muscle lipid uptake upon infection, during which Upd3 expression in plasmatocytes is markedly elevated (Péan et al., 2017). A similar mechanism may be observed in larvae infested by wasp parasitoids, in which Upd3-induced JAK-STAT signaling in muscles is essential for an effective immune response (Yang et al., 2015). This may indicate that muscle insulin resistance is essential to effectively combat wasp parasitic infestation. However, in their follow-up study, Yang and Hultmark (2017) showed that insulin signaling in muscles, in contrast to fat body and plasmatocytes, is essential for the effective encapsulation of invaders. Muscle-specific knockdown of insulin receptor resulted in reduced resistance to infection and encapsulation rate. However, these effects can be explained by developmental defects caused by changes in feeding behavior and subsequent malnutrition, as this experimental treatment was induced throughout the life of individuals. Nonetheless, this publication nicely depicts the impact of experimentally induced muscle insulin resistance on systemic carbohydrate metabolism.

The impact of eAdo on decreased energy consumption by non-immune tissues has also been described in *Drosophila* larva upon wasp infestation. eAdo released by activated immune cells silences consumption of C^14^-labeled glucose by virtually all non-immune tissues, leading to decreased growth of imaginal wing discs and delayed metamorphosis. Consequently, this mechanism allows the glucose uptake by immune cells to be increased up to threefold. These effects were mediated by eAdo activation of the adenosine receptors in target tissues (Bajgar et al., 2015).

Based on the aforementioned data, we may say that the effects of macrophage-derived SIFs are dual. They induce nutrient mobilization from central storage organs and concurrently limit their consumption by non-immune tissues and physiological processes. While these effects are essential for the acute-phase response to infection, they may cause nutrient waste and cachexia if activated chronically (Figure 3).

## Immune Cell-Mediated Metabolic Changes Are Not Always Beneficial Upon Infection

Immune cell-derived SIFs increase the titer of circulating carbohydrates and lipids, which are then available to be exploited by the immune system. Subsequently, these nutrients are utilized by activate phagocytes to feed the suddenly increased energy and nutritional demands. Thus, we may presume that this signaling is important for resistance to infection.

Indeed, experimental knockdown of ImpL2 and Upd3 in infection-activated plasmatocytes or systemic abrogation of adenosine signaling pathway leads to the reduced ability of plasmatocytes to fight the pathogens. That manifests in decreased resistance to bacterial infection accompanied by elevated pathogen load in these individuals (Agaisse et al., 2003; Bajgar and Dolezal, 2018; Krejčová et al., 2020). Further studies suggested that such a decrease in resistance to infection is due to reduced availability of nutrients for immune cells. Notably, a mere twofold increase in glucose concentration in fly diet is sufficient to rescue phenotypes caused by a lack of eAdo signaling (Bajgar et al., 2015).

Although SIF signaling is essential for an adequate immune response to acute bacterial infection, it may become maladaptive under certain conditions. Since SIFs mobilize sources primarily for the needs of phagocytes, they may be exploited by the bacteria growing intracellularly. It is well documented that many intracellular pathogens affect the metabolic profile of macrophages to be literarily nourished by the host cell (Teng et al., 2017). Indeed, it has been described for each of the SIFs discussed here that their effects have become maladaptive upon infection with intracellular pathogens such as *Listeria monocytogenes* or *Mycobacterium tuberculosis* (Péan et al., 2017; Bajgar and Dolezal, 2018; Krejčová et al., 2020).

Not only the type of bacterial threat but also the duration of SIF action seems to be central. Prolonged SIF production leads to uncontrolled wasting of nutrients, cachexia, and irreversible damage of tissues silenced by insulin resistance. Indeed, for instance, the production of eAdo by plasmatocytes has to be time-restricted by eAdo degrading enzyme Adenosine deaminase-related growth factor A (Adgf-A). Interestingly, this enzyme is produced by plasmatocytes as well, with an 8 h delay after adenosine. Lack of adgf-A function leads to wasting of glycogen stores and slow-down of development (Bajgar and Dolezal, 2018).

Also, the ImpL2 production by plasmatocytes must be time-restricted. Chronically increased ImpL2 production by plasmatocytes leads to developmental malformations, reduced body size of the individual, and excessive melanization of immune cells (Krejčová et al., 2020). Moreover, the overproduction of ImpL2 causes insulin resistance and cachexia in the *Drosophila* cancer model (Figueroa-Clarevega and Bilder, 2015; Kwon et al., 2015).

Although eAdo, ImpL2, and Upd3 meet the criteria of a selfish immune factor released by the *Drosophila* plasmatocytes, analogous signaling in mammals remains controversial. However, all of these factors have their signaling counterparts in mammals. While the Upd3 functional homolog has been identified as IL6, studied mostly for its signaling and metabolic effects in immune response, the ImpL2 mammalian putative functional homolog IGFBP7, known for its ability to attenuate insulin signaling, has not yet been explored in the context of infection. Therefore, we speculate about the evolutionary conservation of the role of these SIFs in the following paragraphs.

## The Function of Immune Cell-Derived Sifs May be Conserved Between Insects and Mammals

Experimental studies performed on insects demonstrate that plasmatocytes release signaling factors to affect systemic metabolism and thus ensure a sufficient supply of resources. Here, we would like to consider the possibility that such a mechanism is also valid for mammals (Figure 3). The connection between aerobic glycolysis in activated phagocytic immune cells and the adjustment of systemic metabolism has been considered for mammals in recent review based mainly on clinical data of chronically ill patients (van Niekerk et al., 2017). Moreover, it may represent the essence of many human diseases, as will be discussed later.

A plethora of cytokines and chemokines are released from activated immune cells upon the adoption of a bactericidal polarization state. These are generally known as “pro-inflammatory cytokines” due to their potential to guide other myeloid cells toward inflammatory polarization. Here, we suggest their role in the regulation of systemic metabolism via the induction of insulin resistance upon bacterial infection.

From several experimental and clinical studies, it is clear that macrophage production of pro-inflammatory cytokines is associated with HIF1α transcriptional activity and subsequent metabolic rearrangement toward aerobic glycolysis (Palazon et al., 2014; Corcoran and O’Neill, 2016). However, it is difficult to distinguish whether their production reflects either cellular metabolic switch or adopted pro-inflammatory state since both are intimately interconnected (Diskin and Pålsson-McDermott, 2018). To solve this problem, we must focus on the production of cytokines by cells utilizing HIF1α-mediated aerobic glycolysis in non-inflammatory context, such as neoplastic tumors and hypoxic tissues (He et al., 2014; Edwardson et al., 2017).

There is a compelling list of publications describing the release of pro-inflammatory cytokines from cancer and hypoxic tissues (Dinarello, 2006; Peyssonnaux et al., 2007; Popa et al., 2007; Heikkilä et al., 2008; Xing and Lu, 2016; Lewis and Elks, 2019; Kammerer et al., 2020). Recently, a transcriptomic meta-analysis of human cancers varying in degree of their pro-cachectic potential has been performed to identify new cachectic factors (Freire et al., 2020). Many of the identified factors were cytokines and chemokines well-known for their participation in the acute immune response. That is in concordance with other studies documenting the pro-cachectic features of Il1β, TNFα, and Il6 (Zhang et al., 2007; Narsale and Carson, 2014; Patel and Patel, 2017).

Consistent with this hypothesis, hypoxic tissues also release a number of cytokines with pro-cachectic properties. Surprisingly, the elicitation of hypoxic response employs several immune-related signaling pathways such as JNK, NF-κB, and Hif1α (Jin et al., 2000; D’Ignazio and Rocha, 2016). Their activation leads to the adjustment of cellular metabolism to overcome periods of mitochondrial dysfunction. Although pro-inflammatory cytokines were originally investigated in the context of LSP-induced sepsis (Pizarro and Cominelli, 2007; Rossol et al., 2011), they also reflect the metabolic status and nutritional requirements of their producers and thus serve as potential regulators of systemic metabolism.

According to the proposed theory, the central mechanism that changes the systemic metabolism from anabolism to catabolism is the induction of insulin resistance. In adipose tissue, the lack of insulin signaling serves as a signal for potentiation of lipolysis and subsequent fatty acid mobilization (Langin, 2013). Therefore, infection-induced lipodystrophy results in a substantial release of lipid stores during the acute phase of the immune response. Deliberated fatty acids are further metabolized in the liver and enwrapped into lipoproteins as a lipid form suitable for transport to distant tissues on the periphery (Perry et al., 2014). The liver is known to respond differently to a lack of insulin signaling from most tissues in the body, which is called the “liver insulin resistance paradox” (Santoleri and Titchenell, 2019). Indeed, contrary to other tissues silenced by a lack of insulin signal, hepatic insulin resistance accelerates lipid synthesis, gluconeogenesis, and absorption of circulating amino acids (Biddinger et al., 2008). All of these metabolic changes lead to increased mobilization of lipoproteins and glucose into circulation, resulting in the development of hyperglycemia and hyperlipidemia (Lewis et al., 2002). It is known that stress-related hyperglycemia, as a result of insulin resistance in critically ill and septic patients, is beneficial under certain conditions. In the acute phase of stress response, hyperglycemia appears to support metabolically stressed tissues and immune cell function, whereas in context of its chronic activation, it may result in development of glucotoxicity, exaggerated glycosylation, and chronic inflammation. The function of mammalian immune cells is affected by insulin signaling with different context-dependent effects (Van den Berghe, 2002; Marik and Bellomo, 2013; van Niekerk et al., 2017).

An opposite effect of insulin resistance can be observed in muscles, where a lack of insulin signal leads to a significant reduction of its metabolic rate and induction of autophagy (Lim et al., 2014; Ryter et al., 2014). Autophagy covers basal nutritional demands of silenced cells and concurrently generates amino acids utilized for gluconeogenesis in hepatocytes (Cui et al., 2019). In line with the energy-saving program, insulin resistance in the brain also significantly reduces its energy consumption, leading to a lower intellectual capacity, bad moods, and depressions (Kullmann et al., 2020). Nevertheless, metabolic adaptation to metabolic stress is a tremendously complex process in mammals, which is affected by many hormonal and signaling cues. Particularly effect of several stress-related hormones, such as cortisol, noradrenaline, or norepinephrine on the mobilization of nutrients from adipose tissue and the liver is well established. In this context, the role of immune cell-derived factors on these signaling pathways should also be considered.

Besides the systemic impact on insulin resistance, we should also take into account the paracrine effects of cytokines in the liver. The liver is the central metabolic organ that coordinates the systemic metabolic changes upon infection (Bernal, 2016). In addition, the liver hosts a specialized population of tissue-resident macrophages known as Kupffer cells (KC). KCs serve as sentinel cells reflecting changes in the titers of metabolites and endotoxins in the blood. Although KCs tolerate some levels of endotoxins being permanently present in the circulation without eliciting an immune response, their increase above a certain limit leads to KC activation (Zeng et al., 2016). KCs recognize endotoxins via TLR4, which in turn leads to the activation of NF-κB and its classical M1 polarization (Gandhi, 2020). This process is accompanied by the stabilization of HIF1α and the adoption of aerobic glycolysis (Roth and Copple, 2015). Subsequently, KCs release the pro-inflammatory cytokines Il1β, TNFα, and IL6 into the extracellular space. Consequently, these signaling factors induce hepatocyte insulin resistance via their paracrine signaling (Bartolomé et al., 2008). The lack of insulin signaling in hepatocytes leads to a nuclear translocation of the transcription factor FOXO and the subsequent induction of its specific transcriptomic program. FOXO increases the expression of genes involved in lipogenesis and glycogenolysis, as well as the production and release of lipoproteins (Puigserver et al., 2003). This mechanism is strikingly reminiscent of the process observed in insect adipose tissue. Although the role of KC-derived IL1β, Il6, and TNFα in inducing hepatocyte insulin resistance has been reliably demonstrated, their mere administration cannot fully mimic the effects of KCs (Bartolomé et al., 2008). This suggests the involvement of additional KC-derived signaling factors. IGFBP7, a mammalian putative functional homolog of *Drosophila* ImpL2, may be a potential candidate (Figure 3).

It has been shown that IGFBP7 expression increases fourfold in the culture of human THP-1 macrophages in response to their exposure to *Streptococcus pneumoniae* (Krejčová et al., 2020). In addition, IGFBP7 expression increases sixfold in response to the exposure of KCs to excessive lipids in obese mice. Subsequently, IGFBP7 induces hepatocyte insulin resistance, hyperlipidemia, and hyperglycemia prior to the production of KC-derived pro-inflammatory cytokines (Morgantini et al., 2019). Although the experimental data connecting the adoption of aerobic glycolysis by KCs to the production of IGFBP7 are missing, we suggest that this mechanism may be relevant for the mobilization of nutrients for immune cells, upon infection. The role of IGFBP7 and IL6 in the induction of insulin resistance and cachexia is further supported by their increased plasma titer in patients suffering from diseases often accompanied by cachexia, such as morbid obesity, cancer, chronic obstructive pulmonary disease, acute kidney diseases, and liver fibrosis (Liu et al., 2015; Gunnerson et al., 2016; Ruan et al., 2017; Martínez-Castillo et al., 2020). Although nowadays IGFBP7 is associated with diseases accompanied by chronic inflammatory and pathological conditions, we suggest that its beneficial role in nutrient mobilization during an acute immune response should also be considered (Figure 3).

## Summary

This review brings the new perspective that systemic insulin resistance represents an essential mechanism for overcoming the acute phase of bacterial infection. Insulin resistance is induced by immune cell-derived cytokines, which are produced as a reflection of their elevated metabolic demands resulting from the adoption of aerobic glycolysis. These cytokines induce both the mobilization of sources from the storage organs and their suppressed consumption by non-immune tissues. Titers of nutrients thus elevate in circulation to be utilized by the activated immune system. While such metabolic adaptation is fundamental for resistance to extracellular pathogens, it may become maladaptive upon infection by intracellular bacteria exploiting phagocyte cellular stores for its own benefits. Although cytokine-induced insulin resistance is beneficial during acute phase response, its chronic activation may progress into the wasting of nutrients and cachexia (Figure 3), which are severe metabolic disorders accompanying several serious diseases. Understanding the adaptive significance of cytokine-induced insulin resistance may therefore provide new insights into these maladies.

Induction of insulin resistance in hepatocytes is central for the progress of obesity and obesity-associated diseases, such as non-alcoholic steatohepatitis, atherosclerosis, and diabetes. According to the presented hypothesis, liver and systemic insulin resistance are induced by chronically adopted aerobic glycolysis in activated liver macrophages. Reversal of macrophage metabolic switch may thus represent a powerful therapeutic strategy.

## Author Contributions

AB, GK, and TD discussed the topic, conceptualized, wrote, and revised the manuscript. GK created the figures. AB and GK captured the microscopy images. All authors contributed to the article and approved the submitted version.

## Conflict of Interest

The authors declare that the research was conducted in the absence of any commercial or financial relationships that could be construed as a potential conflict of interest.
